# Long non-coding RNA expression profiling in the NCI60 cancer cell line panel using high-throughput RT-qPCR

**DOI:** 10.1038/sdata.2016.52

**Published:** 2016-07-05

**Authors:** Pieter Mestdagh, Steve Lefever, Pieter-Jan Volders, Stefaan Derveaux, Jan Hellemans, Jo Vandesompele

**Affiliations:** 1 Center for Medical Genetics, Ghent University, 9000 Ghent, Belgium; 2 Cancer Research Institute Ghent (CRIG), 9000 Ghent, Belgium; 3 Biogazelle, 9052 Zwijnaarde, Belgium

**Keywords:** Long non-coding RNAs, Reverse transcription polymerase chain reaction, Cancer genomics, High-throughput screening

## Abstract

Long non-coding RNAs (lncRNAs) form a new class of RNA molecules implicated in various aspects of protein coding gene expression regulation. To study lncRNAs in cancer, we generated expression profiles for 1707 human lncRNAs in the NCI60 cancer cell line panel using a high-throughput nanowell RT-qPCR platform. We describe how qPCR assays were designed and validated and provide processed and normalized expression data for further analysis. Data quality is demonstrated by matching the lncRNA expression profiles with phenotypic and genomic characteristics of the cancer cell lines. This data set can be integrated with publicly available omics and pharmacological data sets to uncover novel associations between lncRNA expression and mRNA expression, miRNA expression, DNA copy number, protein coding gene mutation status or drug response

## Background & Summary

Genome-wide studies have shown that the human genome is pervasively transcribed, resulting in the identification of tens of thousands of long non-coding RNA (lncRNA) genes. Many lncRNAs are associated with disease-linked SNPs or show pronounced tissue-specific expression profiles, hinting at a possible role in human disease and development. The potential importance of lncRNAs (and ncRNAs as a whole) in development is further supported by the intriguing observation that organismal complexity (using the number of distinct cell types as a proxy) is strongly correlated to the proportion of the genome that is non-coding^1^. During the last few years, a growing number of studies provided evidence that lncRNA expression is deregulated in cancer and contributes to many of the established cancer hallmarks (e.g. sustained proliferation, evading apoptosis, invasion and metastasis, angiogenesis, genome instability, …)^2^. Notable examples are *HOTAIR* and *MALAT1* that promote metastasis in breast and lung cancer^3,4^, *PCAT-1* driving proliferation in prostate cancer^5^, SAMMSON sustaining energy metabolism in melanoma^6^ and *aHIF* regulating angiogenesis in various cancer types^7^.

**Figure 1:**
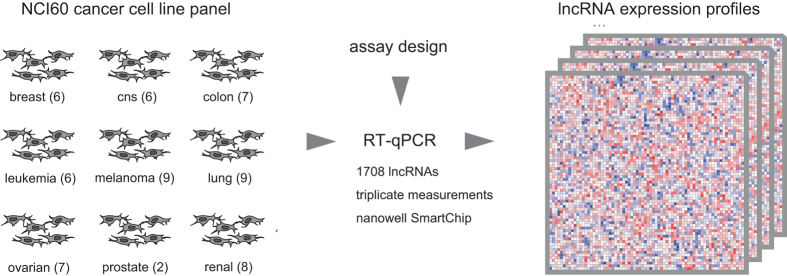
Data generation workflow. RNA isolated from NCI60 cell lines was reverse transcribed and profiled using 1707 lncRNA assays on a SmartChip system.

To further study the role of lncRNAs in cancer, we measured the expression of 1707 human lncRNAs in the NCI60 cancer cell line panel using a high-throughput nanowell RT-qPCR platform (SmartChip, Wafergen) with custom qPCR assays (Fig. 1). These assays were designed using an in-house primer design pipeline (primerXL), taking into account a series of *in silico* quality control steps to ensure optimal assay performance in terms of specificity and efficiency. Upon empirical validation of assay performance, lncRNA expression was quantified in all 60 cancer cell lines followed by data processing and normalization.

The NCI60 cancer cell line panel is one of the best characterized cell line panels for which various omics data sets (including mRNA and miRNA expression, DNA copy number and cancer gene mutation) and drug response data are publicly available. Researchers can reuse the lncRNA expression data described here to study associations with any of the above-described omics data and drug response data and generate hypotheses on lncRNA functionality in cancer.

## Methods

### qPCR assay design and validation

Target lncRNA sequences were obtained from ensembl (v61 and v63) and lncRNAdb (v1) (www.lncRNAdb.org). Whereas lncRNAdb only contains lncRNAs with validated functions, ensembl is not enriched for functional lncRNAs. LncRNA selections from ensembl were random. For lncRNAs overlapping (sense or antisense) with protein coding genes, only the non-overlapping lncRNA sequence was considered for assay design. Detailed annotation of selected lncRNA sequences is available in Supplementary Table 1.

Assays were designed using the primerXL pipeline (www.primerxl.org), applying stringent design criteria to ensure optimal assay performance. Primer annealing sites were evaluated for the presence of SNPs and secondary structures (UNAFold^8^). Assay specificity was evaluated using BiSearch^9^. Assays devoid of SNPs and secondary structures in primer annealing sites with no predicted amplification of non-target sequences were selected for validation.

All assays were validated by means of a titration-response experiment using the MAQC samples as reported previously^10^. In brief, total human reference RNA (MAQC A, Agilent Technologies) and human brain RNA (MAQC B, Ambion) are combined in a 3:1 or 1:3 ratio to create MAQC C and MAQC D respectively. Titration response is calculated for each assay in function of the measured expression difference between MAQC A and MAQC B. In case expression in MAQC A>MAQC B, an assay is considered ‘titrating’ if MAQC A>MAQC C>MAQC D>MAQC B. In case expression in MAQC B>MAQC A, an assay is considered ‘titrating’ if MAQC B>MAQC D>MAQC C>MAQC A.

For a subset of 92 (single exonic) assays we determined amplification efficiency by means of an amplicon dilution series. Amplicons were generated by performing a qPCR reaction with each of the 92 assays on human genomic DNA (Roche). Amplicons were pooled and a 10-fold dilution series ranging from 2.10^7^ to 2.10^1^ molecules was generated using 5 ng l^−1^ carrier RNA. Efficiencies were calculated by combining amplicon concentrations and corresponding assay Cq-values in the following formula: E=(10^−1/S^ −1)×100 with s=slope of the linear regression of log10 (concentrations) and Cq-values.

### RNA isolation and reverse transcription

Cell pellets for each of the NCI60 cancer cell lines were obtained from the National Cancer Institute (Developmental Therapeutics Program). RNA was isolated using the miRNeasy mini kit (Qiagen) according to the manufacturer’s instructions. RNA concentration was measured using the Nanodrop spectrophotometer. RNA from the NCI60 cancer cell line panel and MAQC samples was reverse transcribed using the iScript SsoAdvanced kit (Bio-Rad) with 4 μg of input RNA according to the manufacturer’s instructions.

### qPCR

A total of 1707 lncRNA assays were spotted in triplicate on a SmartChip (Wafergen) to obtain a final concentration of 250 mM primer in a 100 nl reaction volume (Wafergen SmartChip Human lncRNA-1 Panel V1.0, cat n° 430-000103). Per sample, one SmartChip was prepared. A reaction mix containing 4 μg of cDNA (20 μl), 340 μl of 2 X SsoAdvanced Universal SYBR Green Supermix (Bio-Rad), 6.8 μl of BSA (Wafergen), 1.36 μl of yeast cocktail (Wafergen) and 311.8 μl H_2_O was dispensed in each well of the SmartChip to obtain a volume of 100 nl per well. SmartChips were analyzed on a SmartChip Real-Time PCR System using the SmartChip qPCR software (version 2.5.3.69).

### Data processing and normalization

Raw Cq-values (Data Citation 1) were filtered based on a detection threshold of 28 PCR cycles, excluding measurements with Cq>28. This cutoff was derived from the miRNA Quality Control study and represents the Cq-value above which reproducible detection is no longer possible^10^. The median Cq-value of triplicate measurements per gene was subsequently used to calculate a sample-specific normalization factor, applying the global mean normalization strategy^11^. Briefly, each Cq-value was normalized by subtracting the mean Cq-value per sample. Normalized Cq-values were multiplied by -1 such that higher values represent higher expression levels (Data Citation 1 and Supplementary Table 2).

## Data Records

Raw and normalized Cq-values resulting from the RT-qPCR measurements of 1707 lncRNAs in the NCI60 cancer cell lines can be found in the Gene Expression Omnibus (Data Citation 1).

## Technical Validation

### Experimental assay validation

**Figure 2:**
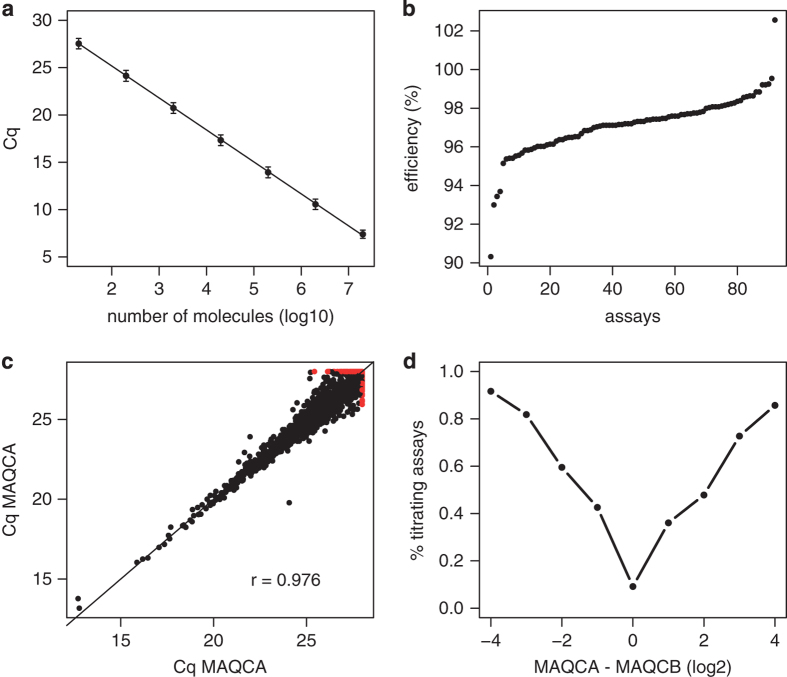
Technical validation of the lncRNA profiling platform. (**a**) Mean Cq-values of 92 lncRNA assays in function of the molecule concentration used in the standard dilution series. Error bars represent the standard deviation of replicate measurements. (**b**) Distribution of efficiencies for all 92 assays. (**c**) Cq-correlation plot for replicate MAQC samples. Assays with Cq-value>28 in one or both replicates were set to 28 and color coded red. (**d**) MAQC titration response curve showing the percentage of titrating assays in relation to the expression difference between MAQCA and MAQCB.

Assay efficiency was determined for a random selection of 92 lncRNA assays using a standard dilution series. Optimal efficiency, generally considered between 90% and 110%, was observed for all tested assays (Fig. 2a,b).

### Assay reproducibility and titration response

Platform reproducibility was validated by replicate measurements of MAQCA RNA (r=0.976, Figure 2c). Assay titration response was evaluated by profiling all four MAQC samples. The percentage of titrating assays was calculated in function of the expression difference between MAQCA and MAQCB and, as expected, increased with an increasing difference between MAQCA and MAQCB (Fig. 2d). The observed titration response was in line with previously reported titration response results for high-throughput RT-qPCR platforms^10^.

### NCI60 lncRNA expression data

**Figure 3:**
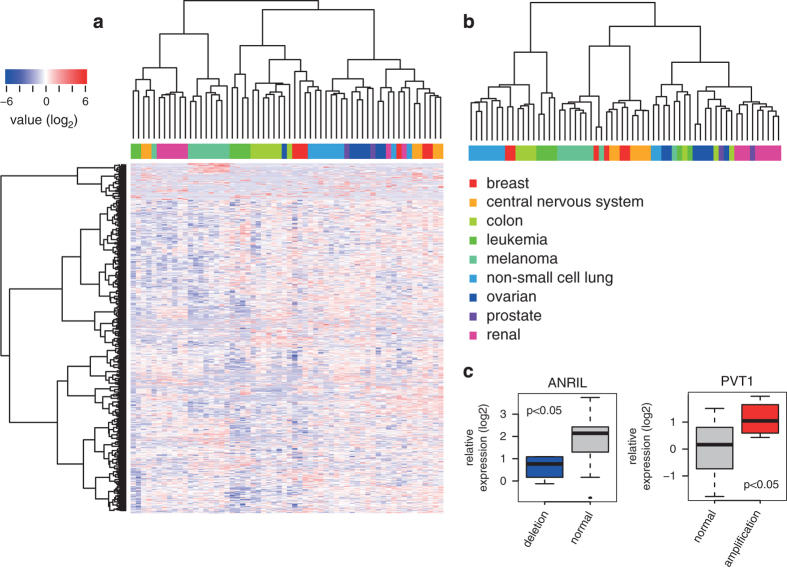
Validation of the NCI60 lncRNA expression data. (**a**) Hierarchical clustering of NCI60 cell lines according to the lncRNA expression data. Cancer types are color-coded. LncRNA expression values are shown as a heatmap. (**b**) Hierarchical clustering of NCI60 cell lines according to public mRNA expression data. (**c**) Expression of ANRIL and PVT1 in cell lines with differential ANRIL and PVT1 copy number.

The NCI60 cancer cell line panel consists of 60 cell lines representing 9 different cancer types. As lncRNAs are known to be differentially expressed between tissue and cancer types^12,13^, lncRNA expression profiles should be able to classify the cell lines in the NCI60 panel. The lncRNA expression profiles in this dataset were filtered to retain only those lncRNAs expressed in at least 80% of the samples per cancer type. Hierarchical clustering of the NCI60 cell lines based on the expression of 1109 lncRNAs showed similar classification accuracy as compared to clustering using expression data from 19,185 mRNAs, obtained from publicly (http://discover.nci.nih.gov/cellminer/) available mRNA expression data (Fig. 3a,b). To further support the technical quality of the NCI60 lncRNA expression dataset, we evaluated the expression of two well-established dosage-sensitive lncRNAs, ANRIL (copy number deletion^14^) and PVT1 (copy number amplification^15^), in relation to their matching copy number status in the NCI60 cell lines. As expected, ANRIL expression was significantly down regulated in cell lines with ANRIL copy number loss whereas PVT1 was significantly up regulated in cell lines with PVT1 copy number amplification (Mann-Whitney *P*<0.05, Fig. 3c). These results demonstrate that the lncRNA expression profiles that have been generated match the phenotypic and genomic characteristics of the cancer cell lines, underscoring the technical quality of the NCI60 RT-qPCR lncRNA expression dataset. Technical quality could also be validated by direct comparison of the RT-qPCR data with matching microarray data obtained from the Cancer Cell Line Encyclopedia. When comparing expression values for 3 well studied lncRNAs (MALAT1, NEAT1 and TUG1), a significant positive correlation between both datasets was observed (Spearman Rank, *P*<0.01). The quality and applicability of this dataset is further exemplified by a recent study in which the expression of the melanoma-specific lncRNA SAMMSON was validated using the NCI60 lncRNA expression profiles presented here^6^.

## Usage Notes

Researchers interested in integrating the lncRNA expression data with matching omics datasets (copy number, mRNA expression, miRNA expression, protein expression, mutation, methylation), drug activity scores or cell line metadata can use the CellMiner webtool (http://discover.nci.nih.gov/cellminer/) to download the respective datasets. Associations between lncRNAs and the above mentioned data layers can be studies through correlation analysis or alternative methods across the 60 cell lines. These include for example correlation analysis between lncRNA and mRNA expression data across cell lines or correlation analysis between lncRNA expression and IC50 values for various compounds across cell lines. Note that lncRNAs are expressed in a more tissue-restricted manner as compared to protein-coding genes, explaining why some lncRNAs only have expression values in a subset of the samples.

## Additional Information

**How to cite this article**: Mestdagh, P. *et al.* Long non-coding RNA expression profiling in the NCI60 cancer cell line panel using high-throughput RT-qPCR. *Sci. Data* 3:160052 doi: 10.1038/sdata.2016.52 (2016).

## Supplementary Material



Supplementary Table 1

Supplementary Table 2
